# Differential associations between traumatic brain injury severity and four dementia phenotypes in military veterans

**DOI:** 10.1002/alz.71370

**Published:** 2026-04-25

**Authors:** Victoria C. Merritt, Elizabeth Valocchi, Francesca V. Lopez, Georgios Asimakopoulos, Alexandra L. Clark, Lay Kodama, Amy J. Jak, Lin Liu, Catherine Chanfreau‐Coffinier

**Affiliations:** ^1^ Research Service VA San Diego Healthcare System San Diego California USA; ^2^ Department of Psychiatry School of Medicine University of California San Diego La Jolla California USA; ^3^ Center of Excellence for Stress and Mental Health (CESAMH) VA San Diego Healthcare System San Diego California USA; ^4^ Department of Psychology The University of Texas at Austin Austin Texas USA; ^5^ Division of Biostatistics and Bioinformatics The Herbert Wertheim School of Public Health and Human Longevity Science University of California San Diego La Jolla California USA; ^6^ Center for the Study of Healthcare Innovation Implementation and Policy (CSHIIP) VA Greater Los Angeles Health Care System Los Angeles California USA

**Keywords:** Alzheimer's disease and related dementias, ADRD, Alzheimer's disease, electronic health records, mTBI, TBI severity, Veterans Affairs

## Abstract

**INTRODUCTION:**

National Veterans Affairs electronic health records were used to examine the association between traumatic brain injury (TBI) severity and four dementia phenotypes ranging from narrow (strict AD) to broad (all‐cause dementia).

**METHODS:**

Veterans with TBI (*n* = 91,753) and a propensity score‐matched comparison group of veterans without TBI (*n* = 183,506) were included. Four validated dementia phenotypes defined using International Classification of Diseases (ICD) codes were evaluated. The association between TBI severity and each dementia phenotype was examined using adjusted logistic regression.

**RESULTS:**

TBI severity was significantly associated with increased odds of developing dementia across the three broader dementia phenotypes in a dose–response manner (moderate/severe/penetrating TBI > mild TBI). Those with unclassified TBI had disease risk falling in between mild and more severe TBI. In contrast, TBI was associated with decreased risk of strict AD across all severity levels.

**DISCUSSION:**

Findings support a dose–response relationship between TBI severity and broader dementia risk but raise questions regarding the TBI–AD link.

## Highlights

- We examined associations between TBI severity and four dementia phenotypes.
- As the dementia phenotype definition broadened, rates of dementia increased.
- TBI history was associated with increased risk of AD+, ADRD, and all‐cause dementia.
- In contrast, TBI history was associated with decreased risk for strict AD.
- Veterans with more severe TBI diagnoses had higher odds of developing dementia.

## 1 BACKGROUND

As life expectancies rise and population age distributions skew older, Alzheimer's disease (AD) and AD and related dementias (ADRD) pose a growing public health challenge. In the United States, an estimated 7.2 million individuals aged 65 and older were living with AD in 2025,^1^ and this number is projected to increase to 13.8 million by 2060.^1^, ^2^ AD/ADRD is a leading cause of dependency and disability among older adults, with health and long‐term care costs estimated at $384 billion in 2025^1^ and projected to grow to $1 trillion in 2050.^1^ These costs do not include unpaid caregiving, which was estimated at 19.2 billion hours and valued at $413.5 billion in 2024.^1^ Given the high prevalence of AD/ADRD and its detrimental effects on patients and caregivers, there has been a great deal of work examining risk and resiliency factors associated with this debilitating disease.

Traumatic brain injury (TBI) has long been hypothesized to be a risk factor for AD/ADRD.^3^, ^4^, ^5^, ^6^ To understand why, it is necessary to have an appreciation of the shared mechanisms that may underlie each condition. Briefly, AD is largely characterized by two hallmark neuropathological features – amyloid plaques and tau tangles.^7^, ^8^ The accumulation of these proteins is ultimately what drives neuronal loss, and this leads to subsequent cognitive and functional decline.^9^ During a TBI, structural damage (e.g., torn vasculature, neuronal death, axonal injury) triggers amyloid and tau pathogenesis within damaged tissue, and secondary cascades involving neuroinflammation (e.g., astrogliosis, microglial activation) and vascular damage (e.g., blood–brain barrier dysfunction, altered cerebral blood flow) may lead to the failed clearance and accumulation of these protein aggregates.^10^, ^11^ These TBI‐initiated neurodegenerative changes may place an individual at increased risk for AD by reducing brain reserve or by exacerbating independent AD‐related processes.

**RESEARCH IN CONTEXT**

1. **Systematic review**: Epidemiological studies examining the association between TBI and dementia have yielded mixed results. These inconsistencies may stem from variation in dementia case definitions and diagnostic algorithms.
2. **Interpretation**: In this national sample of veterans, TBI history was associated with increased odds of disease risk across broader dementia phenotype definitions in a dose–response manner (more severe TBI > mild TBI). In contrast, TBI was associated with decreased odds of developing strict AD. Findings highlight that the strength and direction of the TBI–dementia association vary by disease definition, helping to reconcile inconsistencies observed in the literature. Examining multiple dementia phenotype definitions within the same cohort may help clarify distinct mechanistic pathways linking TBI to heterogeneous neurodegenerative conditions falling within the dementia umbrella.
3. **Future directions**: Additional research is needed to examine TBI severity alongside comorbidities that are common among veterans and to promote greater standardization of dementia classification across studies.

Although several studies have linked prior TBI with an increased risk of AD/ADRD and/or all‐cause dementia,^3^, ^4^, ^6^, ^12^, ^13^, ^14^, ^15^, ^16^ not all studies have found a significant association between the two.^17^, ^18^, ^19^, ^20^ Systematic reviews and meta‐analyses examining these associations further highlight the complex nature of these relationships.^21^, ^22^, ^23^, ^24^, ^25^ Inconsistent findings across studies may be due to widespread methodological heterogeneity, particularly in the definitions and measurements of both TBI and AD/ADRD/dementia. For example, studies vary in their consideration and classification of TBI severity and the ascertainment methods used to identify TBI and AD/ADRD/dementia.^26^ Furthermore, clinical and research diagnostic criteria for AD are continually evolving,^27^, ^28^, ^29^, ^30^, ^31^, ^32^, ^33^, ^34^ and neuropathological studies have revealed that mixed dementia pathologies are incredibly common.^1^, ^35^

This study sought to address these challenges by leveraging nationwide data from the Veterans Health Administration (VHA) to examine the association between TBI severity and four “dementia‐related” phenotypes, ranging from narrow (strict AD) to broad (all‐cause dementia). We were specifically interested in determining whether the association between TBI and dementia varies depending on how the disease outcome is defined.

## 2 METHODS

### 2.1 Procedures

This study was reviewed and approved in 2023 by the VA San Diego Healthcare System Institutional Review Board as well as the US Army Medical Research and Development Command, Office of Human and Animal Research Oversight, Office of Human Research Oversight. This retrospective cohort study utilized electronic health record (EHR) data from the VHA, the largest integrated healthcare system in the United States, serving over nine million veterans annually across 170 VA medical centers.^36^ All EHR data for this study were accessed through the VA Informatics and Computing Infrastructure.^37^

### 2.2 Data sources

#### 2.2.1 Traumatic brain injury

*TBI definition*: TBI diagnosis and severity were determined using a comprehensive list of International Classification of Diseases, Ninth Revision, Clinical Modification (ICD‐9‐CM) and ICD‐10‐CM codes derived from the Armed Forces Health Surveillance Branch (AFHSB) of the Military Health System^38^ (see Table S1a‐S1b for the complete list of ICD‐9/10 codes used). TBI severity was classified as mild, moderate, severe, penetrating, or unclassified according to AFHSB criteria for TBI severity. TBI exposure was defined as the presence of at least one inpatient or two outpatient records with an ICD code for TBI. No TBI exposure was defined as having at least one VHA medical encounter (as evidenced by at least one inpatient or outpatient diagnosis) but no ICD codes for TBI.

Given sample size considerations, veterans with TBI history were categorized into three TBI severity levels for analysis: mild (“mTBI”), moderate/severe/penetrating (“mod/sev/pen TBI”), or unclassified. If a patient had received diagnoses of different severity levels, they were assigned to the most severe TBI category (mod/sev/pen TBI > mTBI > unclassified). For example, if a patient had diagnosis codes falling in the moderate *and* mild TBI range, they were classified as “mod/sev/pen TBI,” and if a patient had diagnosis codes falling in the mild *and* unclassified range, they were classified as “mTBI.”

*Index date and age at index*: For veterans with a history of TBI, the index date was the date of the first TBI diagnosis code documented in the EHR. For veterans with multiple TBI ICD codes of different severity levels, the index date was defined for the most severe TBI. For veterans with no history of TBI, the index date was a randomly assigned date falling between the veteran's first VHA visit date and outcome date (defined below). For all patients, age at index was calculated using the index date and veterans’ date of birth.

#### 2.2.2 Dementia

*Dementia definitions*: Four nested dementia phenotypes were defined using validated ICD code‐based algorithms^39^: (1) “AD” (i.e., strict AD), which includes AD‐specific ICD codes; (2) “AD+,” which includes AD and non‐specific dementia; (3) “ADRD,” which includes AD and related dementias such as vascular dementia; and (4) “all‐cause dementia,” which includes AD, non‐specific dementia codes, related dementias, and other dementias (see Table S2 for the set of ICD codes included in each phenotype). To be classified as having dementia at any level, a veteran needed at least two qualifying ICD codes for that level, recorded on different dates. Absence of dementia was defined as having no ICD codes for any dementia‐related condition, no ICD codes for mild cognitive impairment, and no documentation of any prescription medication for AD. Note that these phenotype definitions were originally developed using VHA EHR data and were tailored to the challenges associated with identifying AD cases within the VHA EHR (see Merritt et al.^39^ for more details).

*Outcome date and age at outcome*: For veterans with a dementia diagnosis, the outcome date was the date of the first dementia ICD code documented in the EHR. For veterans without dementia, the outcome date was the most recent visit date documented in the EHR as of September 30, 2025. For all patients, age at outcome was calculated using the outcome date and date of birth.

#### 2.2.3 Other characteristics

Sex and self‐reported race and ethnicity were extracted from the EHR. Sex was categorized as male and female; race was categorized as American Indian or Alaska Native; Asian or Pacific Islander; Black; White; and Unknown; and ethnicity was categorized as Hispanic/Latino, Not Hispanic/Latino, and Unknown. Other pertinent dates were also extracted from the EHR, including age at first VHA visit, first VHA visit date (year), and observation period (defined as time [in years] receiving VA care, from the first VHA visit to the outcome date).

### 2.3 Participants and study eligibility

Participants (*N* = 275,259) included VHA‐enrolled veterans with a recorded TBI diagnosis between October 1, 1999 to September 30, 2025 and a matched comparison group with no TBI history (defined as veterans with no record of any TBI diagnosis codes in inpatient and outpatient care) receiving VHA care during the same time period.

**FIGURE 1:**
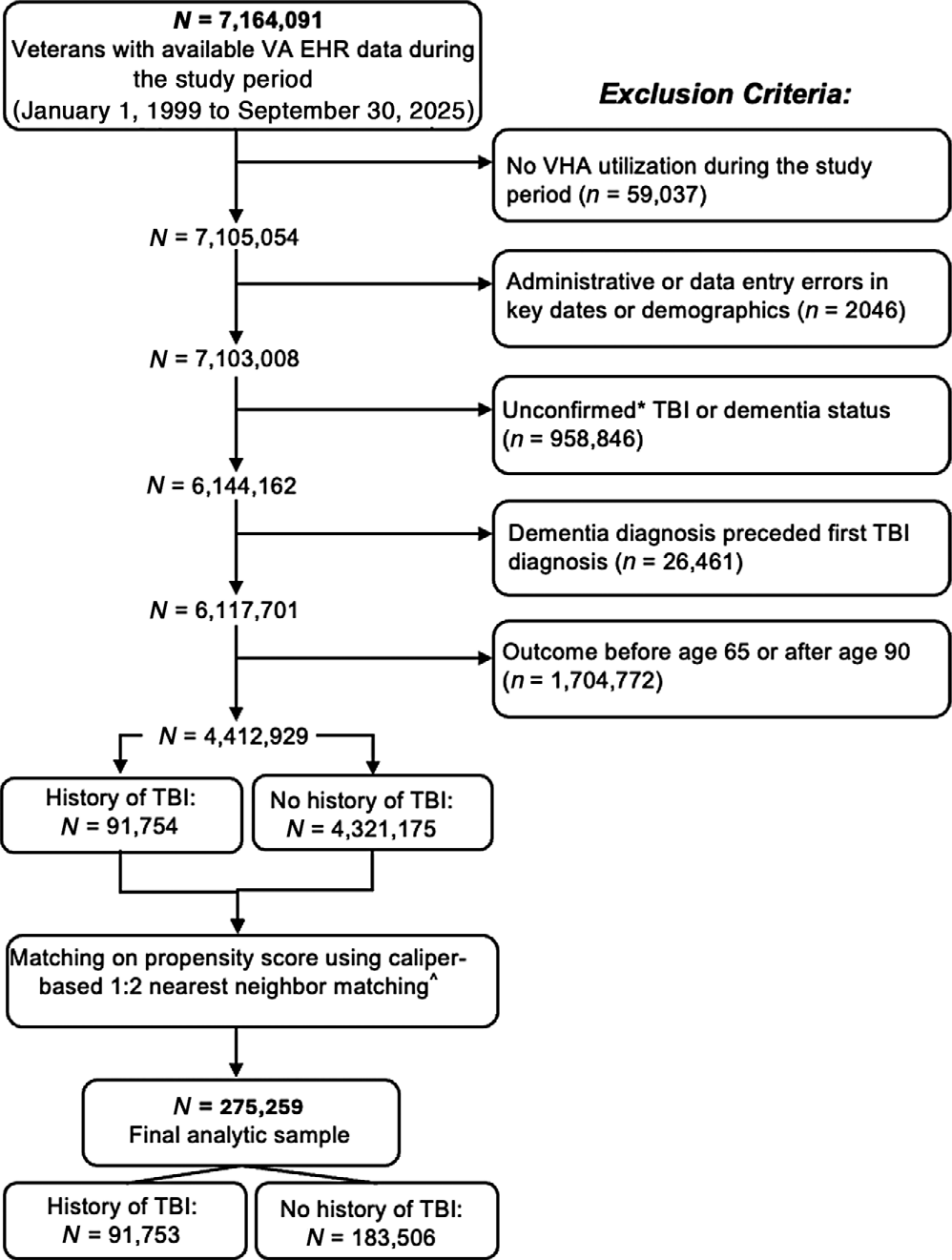
CONSORT diagram for final analytic sample (*N* = 275,259). * Those with an “unconfirmed” TBI or “unconfirmed” dementia status (at any of the four AD/dementia phenotyping levels) were veterans who did not meet the stated definition for the presence or absence of TBI or dementia (e.g., veterans with only one TBI‐related ICD code or those with only one dementia‐related ICD code; see “Methods” for more details). ^ Propensity scores were estimated via logistic regression on first VHA visit year, age at first VHA visit, observation period, sex, race, and ethnicity. Exact matching was applied for sex, race, and ethnicity, and a caliper width of 0.2 standard deviations of the logit of the propensity score was applied to the continuous variables. Veterans with and without a TBI history were matched on propensity score using 1:2 nearest neighbor matching. CONSORT, Consolidated Standards of Reporting Trials; ICD, International Classification of Diseases; TBI, traumatic brain injury; VHA, Veterans Health Administration.

See Figure 1 for a Consolidated Standards of Reporting Trials (CONSORT) diagram outlining the cohort selection procedures. Briefly, the starting VHA EHR cohort included over seven million veterans (*N* = 7,164,091). Veterans were then excluded based on the following criteria: (1) there was no evidence of VHA utilization, defined as the absence of inpatient or outpatient diagnoses in the EHR, during the study period; (2) there were administrative/data entry errors related to pertinent dates or demographics (e.g., date of birth was after first VHA visit date; first diagnosis date was before first VHA visit date); (3) TBI status or dementia status could not be determined (i.e., TBI or dementia status was “unconfirmed”); (4) they had a dementia diagnosis that preceded a TBI diagnosis; and (5) dementia diagnosis (or most recent visit date) occurred before age 65 or after age 90.

Those with an “unconfirmed” TBI status were veterans who did not meet the stated definition of TBI exposure or no exposure (i.e., a veteran with only one TBI‐related ICD code was considered “unconfirmed” because they could not be clearly assigned a TBI exposure status). Similarly, those with an “unconfirmed” dementia status (at any of the four AD/dementia phenotyping levels) were veterans who did not meet the stated definition for presence or absence of dementia (i.e., veterans with only one dementia‐related ICD code was considered “unconfirmed” because they could not be assigned a clear outcome status).

After applying the above exclusion criteria, over four million veterans were retained (*N* = 4,412,929). Propensity score matching was then performed to balance veterans with and without a history of TBI on baseline characteristics. The propensity scores were generated using a logistic regression model including first VHA visit year (to account for differences across the sample regarding the year in which they entered the VA and began receiving care), age at first VHA visit, observation period (i.e., years receiving VA care), sex, race, and ethnicity; 1:2 nearest‐neighbor matching without replacement was performed using a 0.2 standard deviation caliper on the logit of the propensity score with exact matching on sex, race, and ethnicity. To assess the validity of the matching, we examined whether the balance of covariates between veterans with and without a history of TBI was improved after matching by comparing each covariate before and after propensity score matching (see Table S3 for complete data). Sample characteristics for the matched and unmatched samples are included in Table S4.

### 2.4 Data analysis

All analyses were performed using R Statistical Software version 4.4.3.^40^ Differences in veteran characteristics between matched TBI exposed and unexposed veterans were evaluated using Wilcoxon rank‐sum tests or Pearson's chi‐squared tests, as appropriate. The association between TBI and each dementia phenotype was examined using logistic regression adjusted for age at index and observation period. Odds ratios (ORs) and 95% confidence intervals (CI's) were reported for all logistic models.

## 3 RESULTS

The analytic sample (*N* = 275,259 veterans) included 91,753 (33.33%) veterans with TBI and 183,506 (66.67%) veterans without TBI after propensity score matching. Age at index was, on average, 66.42 years (standard deviation [SD] = 9.46). Veterans were predominantly male (95.39%), White (73.33%), and non‐Hispanic (88.85%). Of the veterans with a TBI history, 35,951 (39.18%) were classified as mTBI, 41,085 (44.78%) were classified as mod/sev/pen TBI, and 14,717 (16.04%) were unclassified. Table 1 summarizes sample characteristics for the overall sample and by TBI group (TBI vs no TBI), and Table S5 includes sample characteristics by TBI severity (i.e., no TBI, mTBI, mod/sev/pen TBI, and unclassified).

**TABLE 1:** Participant characteristics for overall sample and by TBI group.

Variables	Overall	No TBI	TBI
	(*N* = 275,259)	(*N* = 183,506)	(*N* = 91,753)
Age at index			
Min, Max	39, 90	39, 90	39, 90
Mean (SD)	66.42 (9.46)	66.40 (9.27)	66.45 (9.83)
Median (Q1, Q3)	66 (60, 73)	66 (60, 73)	66 (60, 73)
Index date (year)			
Min, Max	1999, 2025	1999, 2025	1999, 2025
Mean (SD)	2013 (7.06)	2013 (7.07)	2013 (7.04)
Median (Q1, Q3)	2013 (2007, 2019)	2014 (2007, 2019)	2013 (2007, 2019)
Age at outcome			
Min, Max	65, 90	65, 90	65, 90
Mean (SD)	74.89 (6.48)	75.20 (6.42)	74.26 (6.56)
Median (Q1, Q3)	75 (69, 79)	75 (70, 79)	74 (69, 79)
Outcome date (year)			
Min, Max	1999, 2025	1999, 2025	1999, 2025
Mean (SD)	2020 (6.42)	2020 (6.60)	2021 (6.03)
Median (Q1, Q3)	2025 (2019, 2025)	2025 (2019, 2025)	2024 (2018, 2025)
Age at first VHA visit			
Min, Max	39, 90	39, 90	39, 90
Mean (SD)	57.07 (9.78)	57.06 (9.71)	57.10 (9.92)
Median (Q1, Q3)	56 (50, 64)	56 (50, 64)	56 (50, 63)
First VHA visit date (year)			
Min, Max	1999, 2025	1999, 2025	1999, 2025
Mean (SD)	2003 (5.47)	2003 (5.42)	2003 (5.57)
Median (Q1, Q3)	2001 (1999, 2006)	2001 (1999, 2006)	2001 (1999, 2007)
Observation period (no. years)			
Min, Max	0, 26	0, 26	0, 26
Mean (SD)	9.35 (6.77)	9.35 (6.74)	9.35 (6.83)
Median (Q1, Q3)	8.51 (3.45, 14.35)	8.52 (3.44, 14.32)	8.50 (3.46, 14.43)
Sex			
Male	262,560 (95.39)	175,040 (95.39)	87,520 (95.39)
Female	12,699 (4.61)	8466 (4.61)	4233 (4.61)
Race			
American Indian or Alaska Native	2592 (0.94)	1728 (0.94)	864 (0.94)
Asian or Pacific Islander	3999 (1.45)	2666 (1.45)	1333 (1.45)
Black	44,367 (16.12)	29,578 (16.12)	14,789 (16.12)
Unknown	22,455 (8.16)	14,970 (8.16)	7485 (8.16)
White	201,846 (73.33)	134,564 (73.33)	67,282 (73.33)
Ethnicity			
Not Hispanic or Latino	244,572 (88.85)	163,048 (88.85)	81,524 (88.85)
Hispanic or Latino	18,033 (6.55)	12,022 (6.55)	6011 (6.55)
Unknown	12,654 (4.60)	8436 (4.60)	4218 (4.60)

Abbreviations: TBI, traumatic brain injury; VHA, Veterans Health Administration.

In the overall sample, the cumulative incidence of dementia by phenotype ranged from 3.15% to 17.21%; as the dementia phenotype definition broadened, cumulative incidence of dementia increased (Table 2). Among veterans with TBI history, cumulative incidence of dementia was 2.59% to 18.63% across the various phenotypes; among veterans without TBI history, cumulative incidence of dementia was 3.43% to 16.50%. For all phenotypes other than strict AD, cumulative incidence of dementia was greater in those with TBI compared to those without TBI (all *p *< 0.001; Table 2). For strict AD, cumulative incidence of disease was greater in those without TBI history compared to those with TBI history (*p *< 0.001; Table 2). Table S6 includes details on the cumulative incidence of dementia as a function of TBI severity.

**TABLE 2:** Dementia rates for overall sample and by TBI group.

Phenotype	Overall (*N* = 275,259)		No TBI (*N* = 183,506)		TBI (*N* = 91,753)		*P*
	*N*	Percentage (%)	*N*	Percentage (%)	*N*	Percentage (%)	
Strict AD	8661	3.15	6289	3.43	2372	2.59	<0.001
AD+	33,463	12.16	21,302	11.61	12,161	13.25	<0.001
ADRD	38,311	13.92	24,107	13.14	14,204	15.48	<0.001
Dementia	47,363	17.21	30,271	16.50	17,092	18.63	<0.001

Abbreviations: AD, Alzheimer's disease; AD+, Alzheimer's disease plus non‐specific dementia; ADRD, Alzheimer's disease and related dementias; Dementia, all‐cause dementia; TBI, traumatic brain injury.

**FIGURE 2 alz71370-fig-0002:**
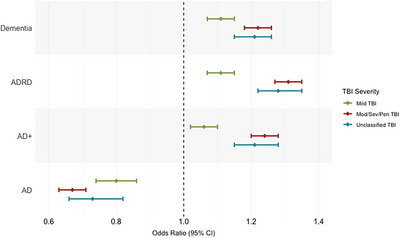
Odds ratios with 95% confidence intervals for each dementia phenotype, adjusted for age at index and observation period. Reference: no history of TBI. AD, Alzheimer's disease; AD+, Alzheimer's disease plus non‐specific dementia; ADRD, Alzheimer's disease and related dementia; CONSORT, Consolidated Standards of Reporting Trials; Mod/Sev/Pen, moderate/severe/penetrating; TBI, traumatic brain injury.

Adjusted logistic regressions evaluating associations between TBI severity and the four dementia‐related phenotypes showed that TBI history of all severity levels was significantly associated with increased risk for dementia across the three broader dementia phenotypes (OR = 1.06 to 1.31; 95% CI = 1.02 to 1.35; Table 3, Figure 2). Specifically, a dose–response relationship of TBI severity with AD+, ADRD, and all‐cause dementia phenotypes was observed, such that those with mod/sev/pen TBI had higher odds of dementia than those with mTBI. Veterans with unclassified TBI also had elevated risk for AD+, ADRD, and all‐cause dementia, with ORs falling between mTBI and mod/sev/pen TBI. In contrast, TBI history of all severity levels was associated with decreased risk for strict AD (OR = 0.67 to 0.80; 95% CI = 0.63 to 0.86).

**TABLE 3:** Associations between TBI severity and dementia phenotypes.

	Strict AD (*n* = 8661)	AD+ (*n* = 33,463)	ADRD (*n* = 38,311)	Dementia (*n* = 47,363)
Mild TBI	0.80 (0.74, 0.86)	1.06 (1.02, 1.10)	1.11 (1.07, 1.15)	1.11 (1.07, 1.15)
Mod/Sev/Pen TBI	0.67 (0.63, 0.71)	1.24 (1.20, 1.28)	1.31 (1.27, 1.35)	1.22 (1.18, 1.26)
Unclassified TBI	0.73 (0.66, 0.82)	1.21 (1.15, 1.28)	1.28 (1.22, 1.35)	1.21 (1.15, 1.26)

*Notes*: Odds ratios (95% CI) from logistic models for the likelihood of each dementia phenotype adjusted for age at index and observation period. Reference: no history of TBI.

Abbreviations: AD, Alzheimer's disease; AD+, Alzheimer's disease plus non‐specific dementia; ADRD, Alzheimer's disease and related dementia; Dementia, All‐cause dementia; Mod/Sev/Pen, moderate/severe/penetrating; TBI, traumatic brain injury.

## 4 DISCUSSION

Using a nationwide[Fig alz71370-fig-0002] sample of VHA‐enrolled veterans, we explored the association between TBI severity and four dementia‐related phenotypes ranging from narrow (strict AD) to broad (all‐cause dementia), with the goal of identifying whether the association between TBI and dementia varied depending on how the disease was defined. Our study revealed three key findings: (1) as the dementia phenotype definition broadened, cumulative incidence of dementia increased in both TBI‐exposed and unexposed veterans; (2) veterans with a history of TBI had a higher cumulative incidence of dementia than veterans without a TBI history across the broader dementia phenotypes, but not the strict‐AD phenotype; and (3) TBI severity was significantly associated with increased odds of developing dementia across the broader dementia phenotypes in a dose–response manner. Specifically, veterans with more severe TBI diagnoses had higher odds of developing AD+, ADRD, and all‐cause dementia compared to veterans with mTBI, and those with unclassified TBI had a disease risk falling in between mTBI and mod/sev/pen TBI. In contrast, TBI was associated with decreased risk for strict AD across all severity levels. Overall, our findings bolster previous research supporting the TBI–dementia link,^41^, ^42^, ^43^ offering further evidence of a dose–response relationship between TBI severity and dementia risk in military veterans. Results also highlight how examining different dementia phenotypes in the same cohort may help to explain some of the inconsistencies observed in the TBI–dementia literature, calling attention to the importance of methodological rigor and transparency in the reporting of outcome variable definitions (i.e., dementia phenotypes). Finally, findings facilitate a better understanding of the possible mechanistic associations between TBI and neurodegenerative conditions falling within the dementia umbrella.

Although many prior studies have examined the association between TBI and dementia, the literature has been hampered by methodological heterogeneity. One notable limitation of prior research is the inconsistent consideration of TBI severity when modeling associations with dementia risk. While some studies specifically sought to evaluate only mTBI,^14^, ^44^ other studies combined all severities into one TBI category and assessed dementia risk across TBI cases and controls.^19^, ^45^ These studies have unsurprisingly reported mixed findings regarding the strength of the association between TBI and dementia. Conversely, when TBI severity was assessed, studies have more consistently demonstrated a significant association between moderate to severe TBI and dementia,^12^, ^15^, ^46^ with some also showing mTBI as a significant risk factor for dementia.^14^, ^15^ The present results add to the growing body of literature supporting a compounding relationship between TBI severity and dementia, that is, as TBI increases in severity, the risk for dementia also increases. We additionally evaluated unclassified TBI, which included veterans with a confirmed history of TBI per ICD codes, but without sufficient detail to determine a severity level – a TBI classification level that has often been excluded in prior studies or simply considered within a mixed TBI case/control framework. Interestingly, a significant association between unclassified TBI and dementia was observed, with its strength of association with dementia generally falling between mTBI and mod/sev/pen TBI, supporting the utility of evaluating and including unclassified TBI in TBI–dementia studies.

Another key challenge associated with prior TBI–dementia research has been the application of wide‐ranging AD/ADRD and dementia definitions and ascertainment methods. To better address this, we evaluated four validated dementia‐related phenotypes within the same cohort using a large, nationwide sample of veterans. Our findings highlight differential associations between TBI severity and the various dementia phenotypes, with notable distinctions between strict AD versus the broader dementia phenotypes. The strongest positive association we observed was between mod/sev/pen TBI and ADRD. Our ADRD definition included AD‐specific ICD codes, non‐specific dementia ICD codes, and related dementia ICD codes (e.g., vascular dementia, Lewy body dementia) and thus captured wide‐ranging pathology. This particular finding is consistent with previous systematic reviews and meta‐analyses that showed a robust relationship between TBI and broader dementia phenotypes.^23^, ^24^

In contrast to ADRD, results showed that TBI (across all severity levels) was associated with *decreased* risk for strict AD. Prior studies examining the relationship between TBI and AD have produced mixed results, with some research demonstrating increased risk for AD in those with a history of TBI^47^ and others showing a lack of association between TBI and AD.^20^, ^48^ While more investigation is needed to better understand the decreased TBI–AD association observed in the present study, we suspect that our counterintuitive finding may, in part, be due to ICD coding practices within the VA. Prior research showed that strict AD was undercoded and underdiagnosed within the VA, as providers tend to utilize non‐specific dementia codes in clinical practice (e.g., “dementia not otherwise specified”),^39^, ^49^ despite AD being the most common cause of dementia.^1^ This coding practice may reflect clinician hesitancy to assign AD‐specific diagnoses due to concerns about stigma or a preference for conservative diagnostic approaches when definitive biomarker confirmation is unavailable. Importantly, the decreased association between TBI and strict AD, combined with the increased associations between TBI and the broader dementia phenotypes, suggests our findings may be more reflective of these diagnostic coding practices as opposed to a true protective effect of TBI on AD pathology. Nevertheless, more research is needed to help clarify whether the observed inverse association reflects coding artifact or true biological distinctions.

Another possible explanation of our findings is that the underlying mechanisms or pathways linking TBI to strict AD may be distinct from the mechanisms associated with TBI and a more broadly defined ADRD/dementia. Indeed, it was previously theorized that the dementia syndromes that emerge following TBI may have a pathology distinct from strict AD, as evidenced by unique clinical presentations,^50^ as well as divergent neuropathology.^51^ Furthermore, TBI has been characterized as a cerebrovascular injury, especially in the case of more severe brain injuries, with evidence of decreased cerebral blood flow following injury, as well as intracranial bleeding (e.g., subarachnoid hemorrhage) and blood–brain barrier disruption – alterations that disrupt the neurovascular unit and potentially lead to long‐term neurodegeneration.^52^ Thus, it is possible that the cerebrovascular damage that occurs following TBI initiates a host of neuropathological processes, which could increase susceptibility for future neurodegeneration, but that the particular neuropathology that ensues may differ depending on the characteristics of the TBI. Finally, the relationship between TBI and AD, compared to TBI and dementia, may depend on other risk factors (e.g., cardiovascular or cardiometabolic health conditions) that emerge as veterans age and that differentially interact with TBI to influence susceptibility for neurogenerative disease.^53^, ^54^

This study has both strengths and weaknesses that should be considered when interpreting the findings. Notable strengths include the use of a large, nationwide sample of veterans that afforded robust power to examine associations between TBI severity and dementia. We also used four dementia‐related phenotypes previously validated in a veteran‐based cohort and strongly associated with AD‐related genetic risk variables (i.e., apolipoprotein E ε4 [APOE ε4] and an AD polygenic risk score),^39^ which allowed for a more comprehensive understanding of the association between TBI and dementia. Additional strengths included the use of propensity score matching for TBI‐exposed and unexposed veterans and the consideration of TBI severity using established VA/Department of Defense guidelines.

Study limitations included the sole reliance on administrative data (i.e., EHR data), which have inherent weaknesses related to accuracy and bias. For example, TBI status and severity were based on ICD codes documented within VHA EHRs; thus, it is possible that we are missing documentation of historic TBI or TBI treated outside of the VHA healthcare system, which could lead to misclassification. Still, this type of misclassification or lack of documentation would tend to fall on the mild end of the TBI severity spectrum, as more severe injuries would likely have been caught and documented in VHA EHRs. Relatedly, selecting the most severe TBI as the index event could also introduce misclassification, especially for participants with multiple injuries. Of note, we did not consider or assess multiple TBIs in this study, which could further influence the observed associations. Other limitations relate to generalizability, as we focused specifically on veterans who were active users of the VHA. Additionally, although reflective of the current VHA patient population, the cohort we evaluated predominantly included non‐Hispanic, White males. Finally, we did not have access to veterans’ education history, nor did we evaluate other military‐related characteristics or medical comorbidities that could influence the observed associations. Future research is needed to more comprehensively characterize associations between TBI and dementia in the context of other comorbidities.

To conclude, in this national sample of veterans, those with a history of TBI had higher rates of dementia than those without a history of TBI across the broader dementia phenotypes. Furthermore, as the dementia definition broadened, cumulative incidence of dementia increased among both those with and without a history of TBI. Finally, TBI history was significantly associated with increased odds of developing AD+, ADRD, and all‐cause dementia, with the strongest associations observed between mod/sev/pen TBI and ADRD. In contrast, TBI of all severities was associated with decreased risk of strict AD. Although our findings bolster previous research supporting a dose–response relationship between TBI severity and dementia, more research is needed to examine the TBI–AD link and understand the extraneous factors influencing this association.

## CONFLICT OF INTEREST STATEMENT

The authors declare no conflicts of interest. Any author disclosures are available in the Supporting Information.

## CONSENT STATEMENT

This study was reviewed by the Institutional Review Board (IRB) and determined to be IRB exempt (category 4); thus, informed consent was waived given secondary use of existing EHR data (and no direct interaction with individuals).

## Supporting information



Supporting Information

Supporting Information
